# Long-Pulsed Nd:YAG Laser for the Treatment of Classic Kaposi Sarcoma

**DOI:** 10.1155/carm/9034642

**Published:** 2025-03-23

**Authors:** Domenico Piccolo, Laura Pieri, Irene Fusco, Tiziano Zingoni, Claudio Conforti, Sabrina Tramontozzi

**Affiliations:** ^1^Novea Skin Center-Dermo Aesthetic Laser Centers, Pescara and L'Aquila, Avezzano, Italy; ^2^Department of Clinical Research and Practice, El.En. Group, Calenzano 50041, Italy; ^3^IDI-IRCCS, Dermatological Research Hospital, Rome, Italy

**Keywords:** case report, Kaposi's sarcoma, Nd:YAG laser, videodermoscopy

## Abstract

**Background:** Classic Kaposi's sarcoma (CKS) is distinguished by nodules and clustered papules over the extremities with persistent edema.

**Aim:** This report examine the results of neodymium-doped yttrium-aluminum-garnet (Nd:YAG) laser for the treatment of numerous nodular symptomatic nodules in CKS patients' lower extremities that are ineligible for systemic therapy.

**Methods:** Each lesion received two sessions Nd:YAG laser treatment performed at 1-month intervals. Pretreatment and follow-up visit (after 3 months) were accompanied by clinical photographs and improvement was assessed by a skilled medical professional by comparing before- and after-treatment photos as well as by looking at patients directly. Videodermoscopy was performed on all lesions.

**Results**: Just one session with long-pulsed Nd:YAG laser proved to be successful, with the immediate coagulation of the lesions and the disappearance of most of them. Every single lesion healed in two to 4 weeks. Only one lesion, the largest one, presented a water blister after treatment, which resolved within a few days. At 2 months follow up, the patient reported total pain relief and full recovery after the symptomatic nodules vanished.

**Conclusion:** Nd:YAG laser could represent a rapid and advantageous therapeutic approach for both early- and advanced-stage CKS patients.

## 1. Introduction

Kaposi's sarcoma (KS) is a vascular tumor caused by human herpesvirus-8 (HHV-8) infection [1]. Particularly, classic KS (CKS) is distinguished by nodules and clustered papules over the extremities with persistent edema.

Therapeutic options for KS include cryotherapy, surgical excision, chemical cauterization, laser, intralesional chemotherapies, radiotherapy, immunomodulatory/antiviral drugs, as well as systemic/local treatments [2, 3].

Systemic treatments are limited due to their side effects, and patients with limited skin disease in their early stages should not receive these aggressive treatments [4].

Treatment for exophytic or hemorrhagic multiple nodular lesions may involve minimal surgery, such as curettage. While surgical excision is an option for some well-circumscribed lesions, it is not advised for many large lesions with ill-defined borders [5].

Antiangiogenic treatments are presently being tested in cancer clinical management at a growing rate, for the treatment of KS in both HIV-positive or -negative patients. However, the effectiveness of these medications is still relatively low, and intrinsic or acquired tumor resistance is common [6].

Numerous laser types have been used, including CO2 laser, Argon laser, Dye laser and Nd-YAG laser with positive clinical outcomes [7, 8].

In light of this, this report examine the results of neodymium-doped yttrium-aluminum-garnet (Nd:YAG) laser for the treatment of numerous nodular symptomatic nodules in CKS patients' lower extremities that are ineligible for systemic therapy.

## 2. Case Presentation

A 75-year-old Caucasian woman who had suffered with CKS for at least 10 years. Both clinical and histopathological results supported the diagnosis.

She had no prior history of immunosuppressive drug use, systemic illness, or organ transplantation. Patient's HIV serologies, complete blood counts, and metabolic panels were within normal ranges. The patient exhibited several vascularized nodules located in the lower limbs or feet, ranging in size from 0.3 to 2 cm.

The patient had not received any further regional or systemic treatments, either concurrently or in the previous 3 months, for the CKS management. Each lesion received two sessions Nd:YAG laser treatment (Again family, DEKA M.E.L.A. Srl, Florence, Italy) and an external skin cooling system (Zimmer, Germany) was included with the study device. A longue pulsed Nd:YAG laser at 1064 nm was applied with the typical handpiece, producing a 5 mm spot on the skin's surface using a standard emission mode single-pass, with a fluence of 100–120 J/cm^2^, vascular pulse [9], which consist of a double pulse (5 + 15 ms with a delay of 10 ms). The two sessions were performed at *t* 1-month intervals.

An endpoint reaction was observed, and the ideal number of pulses and interval (milliseconds) between pulses were calculated by increasing the fluence by 10 J/cm^2^ in the larger lesions: the nodular lesions turned grey whitening and made a loud popping sound, indicating that the treatment had been successful.

No local anaesthetic was administered before the laser therapy. During the procedure, patients were instructed to report any severe pain or discomfort they experienced.

After the procedure, a mixture of dexpanthenol and chlorhexidine was administered to the treated areas for 10 days to promote wound healing and protect them from microbial contamination. Possible adverse effects of the treatment were warm sensations or a mild redness around the lesions. The development of blisters, burns, and crusts was also monitored.

Clinical measures were used to evaluate the patients' response to treatment, including flattening of elevated lesions, reduction in the total number of lesions, and reduction in the diameter of representative lesions. Pretreatment and follow-up visit (after 3 months) were accompanied by clinical photographs and improvement was assessed by a skilled medical professional by comparing before- and after-treatment photos as well as by looking at patients directly. Videodermoscopy was performed on all lesions using the FotoFinder System (GmbH 1000, Bad Birnbach, Germany).

Treatment with long-pulsed Nd:YAG laser was successfully applied to several nodular lesions in the upper and lower extremities. Just one session proved to be successful, with the immediate coagulation of the lesions and the disappearance of most of them.

**Figure 1:**
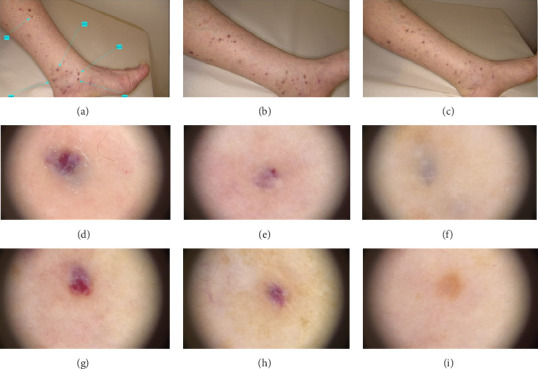
Clinical image of a 75-year-old woman with classic Kaposi's sarcoma who exhibited several vascularized nodules located in the lower limbs before (a), at 1 month after the first laser treatment (b) and at 1 month after the second laser treatment (c). The green arrows with relative number indicate the nodules treated. The corresponding dermatoscopic analysis was carried out for nodule number 3 before (d), at 1 month after the first laser treatment (e) and at 1 month after the second laser treatment (f), and for nodule number 4 before (g), at 1 month after the first laser treatment (h) and at 1 month after the second laser treatment (i).

**Figure 2:**
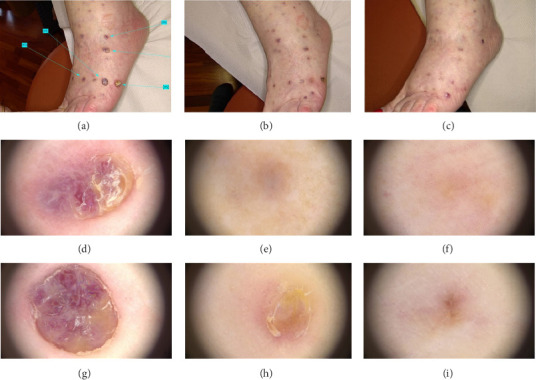
Clinical image of a 75-year-old woman with classic Kaposi's sarcoma who exhibited several vascularized nodules located in the lower limbs before (a), at 1 month after the first laser treatment (b) and at 1 month after the second laser treatment (c). The green arrows with relative number indicate the nodules treated. The corresponding dermatoscopic analysis was carried out for nodule number 5 before (d), at 1 month after the first laser treatment (e) and at 1 month after the second laser treatment (f), and for nodule number 6 before (g), at 1 month after the first laser treatment (h) and at 1 month after the second laser treatment (i).

**Figure 3:**
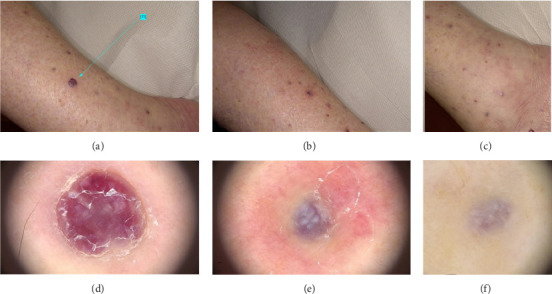
Clinical image of a 75-year-old woman with classic Kaposi's sarcoma who exhibited several vascularized nodules located in the lower limbs before (a), at 1 month after the first laser treatment (b) and at 1 month after the second laser treatment (c). The green arrows with relative number indicate the nodules treated. The corresponding dermatoscopic analysis was carried out for nodule number 11 before (d), at 1 month after the first laser treatment (e) and at 1 month after the second laser treatment (f).

Patient' clinical findings before and after treatment are shown in Figures 1, 2, 3.

During treatment, all lesions, particularly the larger ones, made a “popping” sound, although no bleeding occurred. Only one lesion, the largest one, presented a water blister after treatment, which resolved within a few days.

At 2 months follow up, the patient reported total pain relief and full recovery after the symptomatic nodules vanished. All of the lesions healed in two to 4 weeks.

After the procedure, the treated area developed a crust that healed in two to 3 weeks with scarring.

Although a few atrophic scars appeared where the lesions had coalesced, patient did not express any serious concerns about this side effect.

The patient experienced a significant reduction in her symptoms; she was able to walk and carry out routine activities without requiring assistance. Except of the mild atrophic scars, only for some lesions, no other side effects such as bleeding, local wound infection, altered sensation, hyper- or hypopigmentation, or formation of excessive granulation tissue occurred.

The patient had improved outcomes, particularly in terms of flattening raised lesions, reducing lesion size and pain; she expressed a great satisfaction at having been able to reclaim an acceptable quality of life, especially without pain.

## 3. Discussion

Nd:YAG laser can effectively destroy the vascular lesions linked to CKS by penetrating deep vascular structures with minimal damage to surrounding tissues [8] offering a safe and efficient alternative for patients unable to receive systemic treatment and with multiple widespread lesions.

Our study provided dermoscopic/clinical evidence of the Nd:YAG laser treatment's prompt, secure, and effective outcomes. This laser was used in one session to treat a lot of nodular lesions in patient with stage 4 CKS. There were no further negative effects, except for a small number of lesions that experienced mild atrophic scarring. Our successful outcomes on CKS skin lesions management match with previous research findings [7–10]. The modulation of cytokine expression may be responsible for this effect [11]. Furthermore, the application of a ‘non-uniform' pulse emission mode [9] highlights the effectiveness of this 1064 nm Nd:YAG laser for the treatment of vascular lesions linked to CKS since it was designed to consider the modification of blood absorption and Met-haemoglobin (Met-Hb) formation that results in an increase of the 1064 nm wavelength absorption.

Indeed, several pulses rather than a single pulse are needed to exploit the Met-Hb production. In this way the energy can be reduced, increasing the surrounding tissue's protection [12, 13]. Although the first pulse (5 ms) heats the blood vessel and promotes the production of Met-Hb, it does not obliterate the blood vessel. A pulse delay is necessary to finish the haeme molecule's conformational shift and transform oxyHb (Hb-O2) and Hb into Met-Hb. In this instance, a 10 ms delay is used. In order to support Met-Hb synthesis, a second longer pulse (15 ms) is administered to maintain the blood vessel's internal temperature between 60°C and 70°C (heating duration comparable to the vessel's thermal relaxation period).

As a result, the laser technique's major advantage is its ability to deliver low energy while protecting the surrounding tissue, which results in rapid vascular clearance with minimal patient's discomfort and few side effects. Nd:YAG laser shows promise, as an adjuvant treatment for systemic therapy, in the management of particularly debilitating skin lesions, with a noticeable improvement in two to 3 weeks and a well-tolerated procedure. Its use could represent a rapid and advantageous therapeutic approach for both early- and advanced-stage CKS patients.

According to our findings, the therapeutic innovation of this laser therapy is represented by the faster healing time obtained by the patient and its ability to treat all types of CKS lesions, including larger ones, for which surgery is not recommended (as mentioned in the Introduction section), achieving the complete disappearance of lesion with just one treatment laser session. A further important aspect of this technology is that the patient experienced no pain after the treatment, and she was able to walk and to carry out routine activities without assistance.

Studies with further patients and longer follow-up will be required to validate this therapy.

## 4. Conclusion

The study findings reveal that the use of Nd:YAG laser with a specific vascular pulse could represent a well-tolerated, rapid and advantageous therapeutic approach for both early- and advanced-stage CKS patients who are ineligible for systemic therapy.

## Data Availability

The data that support the findings of this study are available from the corresponding author upon reasonable request.
